# Nandrolone Decanoate and Swimming Affects Cardiodynamic and Morphometric Parameters in the Isolated Rat Heart

**DOI:** 10.3390/life12081242

**Published:** 2022-08-16

**Authors:** Jasmina Sretenovic, Vladimir Zivkovic, Ivan Srejovic, Suzana Pantovic, Jovana Joksimovic Jovic, Maja Nikolic, Tamara Nikolic Turnic, Maja Savic, Maja Jevdjevic, Zoran Milosavljevic, Sergej Bolevich, Vladimir Jakovljevic

**Affiliations:** 1 Department of Physiology, Faculty of Medical Sciences, University of Kragujevac, Svetozara Markovica 69, 34000 Kragujevac, Serbia; 2 Department of Pharmacology 1st Moscow State Medical University IM Sechenov, Trubetskaya Str. 2, 119992 Moscow, Russia; 3 Department of Pharmacy, Faculty of Medical Sciences, University of Kragujevac, Svetozara Markovica 69, 34000 Kragujevac, Serbia; 4 N.A. Semashko Public Health and Healthcare Department, F.F. Erismann Institute of Public Health, I.M. Sechenov First Moscow State Medical University, 119435 Moscow, Russia; 5 Department of Histology and Embryology, Faculty of Medical Sciences, University of Kragujevac, Svetozara Markovica 69, 34000 Kragujevac, Serbia; 6 Faculty of Farmacy, Pavlovica Put bb., Bijeljina University, 76300 Bijeljina, Bosnia and Herzegovina; 7 Department of Human Pathology, 1st Moscow State Medical University IM Sechenov, Trubetskaya Str. 2, 119992 Moscow, Russia

**Keywords:** isolated rat heart, cardiodynamics, nandrolone decanoate, morphometry, collagen

## Abstract

(1) Background: The aim of this study was to show the effects of swimming and nandrolone administration on cardiodynamic and morphometric parameters of the isolated rat heart. (2) The study included 72 Wistar rats, divided into three groups, scheduled to be sacrificed after the second, third, and fourth week. Each group was divided into four subgroups: control (T-N-), nandrolone (T-N+), swimming training (T+N-), and swimming training plus nandrolone (T+N+) group. The rats from T+N- and T+N+ swam 1 h/day, 5 days/week while ones from T-N+ and T+N+ received weekly nandrolone decanoate (20 mg/kg). The isolated hearts were perfused according to the Langendorff technique and measured parameters: dp/dt max/min, SLVP, DLVP, heart rate, and coronary flow. Hearts were fixed and stained with H/E and Masson trichrome dyes. (3) dp/dt max and dp/dt min were increased in the T-N+ group at higher perfusion pressure compared to the T-N- group. SLVP and DLVP were increased in all groups after the 4th week. Collagen content was increased in T-N+ by 403% and in T+N+ by 357% groups, while it was decreased in T+N- compared to the control after 4th week. (4) Conclusions: Nandrolone alone or combined with swimming had a deleterious effect on myocardial function and perfusion.

## 1. Introduction

Anabolic androgenic steroids (AAS) belong to a large group of synthetic derivatives of the male sex hormone testosterone. They were created to maximize anabolic and minimize androgenic effects [1]. It is known that AAS are used to treat various medical conditions, such as osteoporosis [2], hypogonadal dysfunction, aplastic anemia, and malnutrition [3]. However, the use of these agents is not always medically justified. Because of their anabolic effects, in recent decades, the abuse of AAS has become widespread among professional and recreational sportsmen [4,5]. They abuse these drugs in order to improve their physical performance [6], strength, potency, and muscle mass [7]. Nevertheless, for athletic enhancement, the individuals take AAS in up to 10 to 100 times higher doses than those for therapeutic reasons, which harmfully affect various organ systems [8].

One of the most popular AAS among professional and recreational athletes is nandrolone decanoate, a derivate of 19-nortestosterone, which is usually applied in the form of depot preparations [9]. Nandrolone is chemically similar to the male sex hormone testosterone, but unlike it, it has increased anabolic and reduced androgenic activity [10]. Nandrolone decanoate is a slow-acting anabolic steroid, designed specifically to increase muscle mass [11]. After injection, nandrolone is slowly eliminated from the blood with a half-life of 6 days [10]. After intramuscular injection, in humans, its effects last up to three weeks [9]. The majority of studies on animal models usually administered nandrolone over 4 weeks [12,13,14,15,16,17], which corresponds to one “steroid cycle” in humans [18].

Previous studies have shown that abuse of AAS causes adverse effects on the liver, such as cholestatic jaundice, peliosis hepatis, hepatocellular hyperplasia, and hepatocellular adenoma [19]. Furthermore, in regard to the male reproductive system, the abuse of these agents may induce testicular atrophy [20], followed by the decrease of the diameter of seminiferous tubules and the increase of the interstitium of the testicles [21], impotence, and benign prostate hypertrophy [20]. Most recently, it has also been shown that nandrolone causes harmful effects on pituitary LH and FSH gonadotrophic cells, reflected by the reduction in volume density and number of cells per mm^2^ [16].

AAS causes powerful effects on left ventricular hypertrophy via the androgenic receptor [22], which is localized in the cytoplasm of cardiomyocytes [1,9]. Previous research has shown that anabolic steroids not only induce myocardial hypertrophy with a disproportional accumulation of extracellular collagen [14,23] but can also lead to abnormalities of the impulse conduction (heart rate) and contractility [24,25]. Some studies have shown that the use of supraphysiological doses of AAS can lead to increased interventricular septum thickness, dilated cardiomyopathy, arrhythmia, heart failure, and sudden cardiac death [3,22,26].

On the other hand, exercise and endurance training may cause a series of changes in the cardiac morphology, including increased left ventricular chamber size, wall thickness, and heart mass [27]. It is well known that exercise reduces the risk of cardiovascular disease by affecting the molecular and cellular remodeling of the heart [28]. Previous studies have shown that regular training leads to a reduction of blood pressure levels [29] but also causes improvement in left ventricular performance and myocardial contractility [30,31]. The mechanism involved in cardiac protection, caused by regular training, is not sufficiently understood. Some of the suggested mechanisms are an increase in antioxidant protection [30], as well as an increase in the amount of heat shock protein (HSP) in myocardium [30]. In addition, the kinds of effects on the myocardium and vascular system that are caused by exercise depend on the type of exercise, its frequency, intensity, and duration [9,24]. These exercise characteristics are of particular importance, while a comprehensive regime of training may also exert a harmful effect on health [32].

There is a lack of data regarding the mutual effects of administration of AAS with or without swimming training, especially in time-dependent administration, on the heart and coronary circulation. Since the abuse of AAS has taken on epidemic proportions [4] our study aimed to assess the effects of supraphysiological doses of nandrolone decanoate (alone or in combination with swimming training), in time-dependent administration, on cardiodynamic parameters, and coronary circulation in the isolated rat heart. Moreover, we also evaluated the morphometric parameters and collagen content in the left ventricle myocardium of those isolated hearts.

## 2. Materials and Methods

### 2.1. Experimental Animals

This study included 72 Wistar albino male rats (10 weeks old, weighing 200–250 g). The rats were obtained from the Military Medical Academy, Belgrade, Serbia. Rats were housed in plexiglass transparent cages (six rats per cage). The room temperature was kept at 25 ± 1 °C with 12:12 h light and dark cycles. Food and water were provided ad libitum. 

The rats were randomly classified into 3 groups, and each group was further divided into 4 subgroups. The first group of animals were sacrificed after two weeks, the second group were sacrificed after three weeks, and the third group was sacrificed after four weeks of experimental period:

1. T-N-—Control group (rats without the administration of nandrolone decanoate and swimming training);
2. TN+—Nandrolone group (rats with the administration of nandrolone decanoate over a period of 2, 3, or 4 weeks, without swimming training);
3. T+N-—Swimming training group (swimming (1 h/day, 5 days per week during a period of 2, 3 or 4 weeks) without the administration of nandrolone decanoate);
4. T+N+—Swimming training plus nandrolone group (swimming (1 h/day, 5 days per week) with the administration of nandrolone decanoate during the 2, 3 or 4 weeks).

The rats from steroid-positive groups received nandrolone decanoate (DECA DURABOLIN^®^, Organon, Holland) administration by subcutaneous injection once per week in doses of 20 mg/kg [13,14,15,16]. The rats from trained-positive groups were swimming in the pool, (dimensions 120 × 80 × 50 cm l/w/h) one hour per day, 5 days per week. The training was carried out with the continuous monitoring of the animals and the temperature of the water environment (37 °C). In order to experience the same experimental conditions, rats from non-trained groups were placed into a separate water tank (depth 5 cm) for five minutes at the same water temperature as well as rats from trained groups, whereas the rats from non-steroid treated groups received sesame oil. 

Rats were sacrificed 48 h after the last swimming training session, that is, the first subgroup of animals was sacrificed after the second week, the second subgroup after the third week, and the third subgroup after the fourth week from the beginning of the experimental protocol. The body weight of the rats was measured before sacrificing. 

Under short-term anesthesia (ketamine 100 mg/kg and xylazine 10 mg/kg), animals were premedicated with heparin as an anticoagulant and sacrificed by cervical dislocation (Schedule 1 of the Animals/Scientific Procedures, Act 1986 UK). After emergency thoracotomy, the hearts were rapidly excised, the aortas were cannulated and retrogradely perfused at gradually increased coronary perfusion pressure (CPP) (40–120 H_2_O), according to the Langendorf technique. The composition of the Krebs–Henseleit perfusate was as follows Mm/L: NaCl 118, KCl 4.7, CaCl_2_•2H_2_O 2.5, MgSO_4_•7H_2_O 1.7, NaHCO_3_ 25, KH_2_PO_4_ 1.2, glucose 11, and pyruvate 2, equilibrate with 95% O_2_ plus 5% CO_2_ and warmed to 37 °C (pH 7.4). Immediately after establishing a normal heart rhythm, the sensor (transducer BS4 73-0184, Experimetria Ltd., Budapest, Hungary) was inserted through the newly damaged left atrium and mitral valve into the left ventricle for continuous monitoring of cardiac function.

By placing the sensor in the left ventricle, the following parameters of myocardial function were continuously registered:

1. Maximum rate of pressure development in the left ventricle (dp/dt max);
2. Minimum rate of pressure development in the left ventricle (dp/dt min);
3. Systolic left ventricular pressure (SLVP);
4. Diastolic left ventricular pressure (DLVP);
5. Heart rate (HR).

Coronary flow (CF) was measured flowmetrically.

### 2.2. Histological and Morphometric Analysis

The isolated rat hearts were first measured, then were halved and the left ventricle wall was fully exposed. The heart samples were routinely fixed in 4% neutral formalin, dehydrated in ethanol (70%, 96%, and 100%), cleared in xylene and embedded in Histowax^®^ (Histolab Product AB, Göteborg, Sweden), and processed for further histological analysis. Sections, 5 µm thick, were stained with H/E for the visualization of tissue structures, and with Masson trichrome staining for the detection of the collagen content. The images of tissue sections were captured with a digital camera attached to the Olympus BX51 microscope. Morphometric analysis was carried out using calibrated Axiovision software (Zeiss, White Plains, NY, USA) as well as with Image Pro-Plus (Media Cybernetics, Rockville, MD, USA) according to our previously described methodology. The measurement of the cross-section area and longitudinal section diameter of the cardiomyocyte (morphometric analysis) was performed on at least 100 cells per animal [14,15]. Results for collagen content were presented in percent, in which control values in every experimental week were assigned as 100% and values from experimental groups represented a percentage of increase or decrease in comparison to the control value [15,21]. 

### 2.3. Statistical Analysis

Statistical calculations were made with the SPSS computer program, version 20.0 (SPSS Inc., Chicago, IL, USA), and data are presented as mean values ± standard deviation (SD). Statistical comparison between groups was used by one-way ANOVA test with the post hoc LSD test analysis for multiple comparisons. *p* values below 0.05 were considered statistically significant.

## 3. Results

### 3.1. Heart Weight (HW) and Heart Weight Body Weight Ratio (HW/BW)

The administration of nandrolone decanoate alone decreased heart weight after the second week by 3.5%, after the third experimental week by 3%, and after the fourth week by 4% in comparison to the control value. Swimming training alone or combined with nandrolone increased the heart weight after the second week by 8% and 15%, respectively, after the third week by 6% and 8%, and after the fourth week by 6% and 9%, respectively, in comparison to the control (Table 1). 

The self-administration of nandrolone decreased heart weight/body weight ratio by 11% after the second experimental week compared to control values. Swimming alone increased HW/BW after the second week by 6%, by 10% after the third and by 17% after the fourth experimental week compared to the control group. The combined administration of nandrolone and swimming increased HW/BW by 8.5% after the 2nd experimental week, for 10% after the 3rd and for 12% after the 4th experimental week compared to control (Table 1).

### 3.2. Cardiodinamic Prameters

#### 3.2.1. Maximum Rate of Pressure Development—dp/dt max 

**Figure 1:**
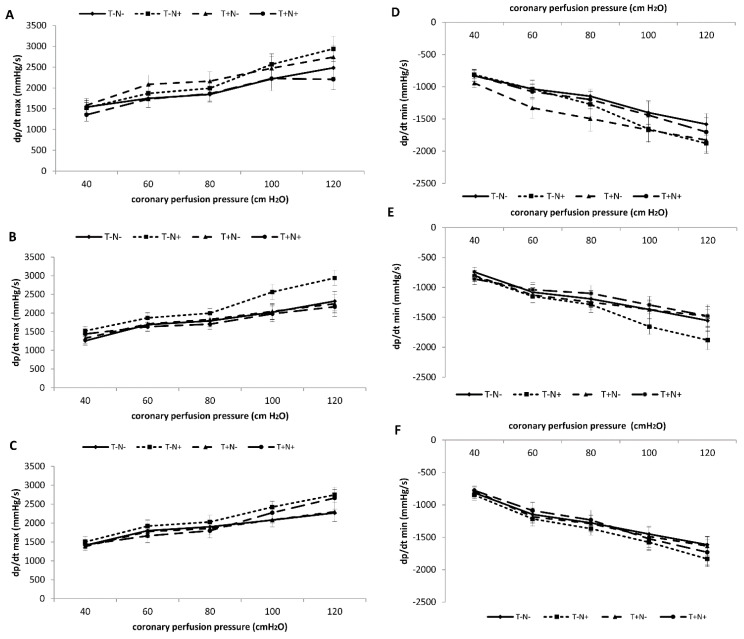
Mean values of dp/dt max (**A**–**C**) and dp/dt min (**D**–**F**). Comparation of all experimental groups the after second (**A**,**D**), third (**B**,**E**), and fourth (**C**,**F**) week of the experimental period. Each value represents the mean ± SD (*n* = 6).

The values of the maximum rate of pressure development in the left ventricle (dp/dt max) after the second week were increased in the T-N+ and T+N- groups, while in the T+N+ group the values were lower at the perfusion pressure 120 cm H_2_O compared to the control group (Figure 1A). By the end of the third week, this parameter was increased in T-N+ in all perfusion pressures compared to other groups (Figure 1B). After the fourth experimental week, nandrolone alone increased dp/dt max in all perfusion pressures compared to all groups, while the combined administration of nandrolone and training increased this parameter in the perfusion pressure starting from 80 to 120 cm H_2_O, compared to control values (Figure 1C). Statistical significances between groups are presented in Table 2.

#### 3.2.2. Minimum Rate of Pressure Development—dp/dt min

After the second experimental week, the largest decrease (more negative values) in dp/dt min was observed in the group which was exposed to training alone compared to the control. Nandrolone alone decreased this parameter in perfusion pressure starting from 80 to 120 cm H_2_O compared to the T-N- (Figure 1D). After the third week of the experiment, nandrolone alone significantly decreased values of dp/dt min, especially at higher perfusion pressures starting from 80 to 120 cm H_2_O compared to the control value (Figure 1E). After the fourth experimental week, values were decreased in the T-N+ group compared to the control (Figure 1F). Statistical differences between groups are presented in Table 2.

#### 3.2.3. Systolic Left Ventricle Pressure—SLVP

**Figure 2:**
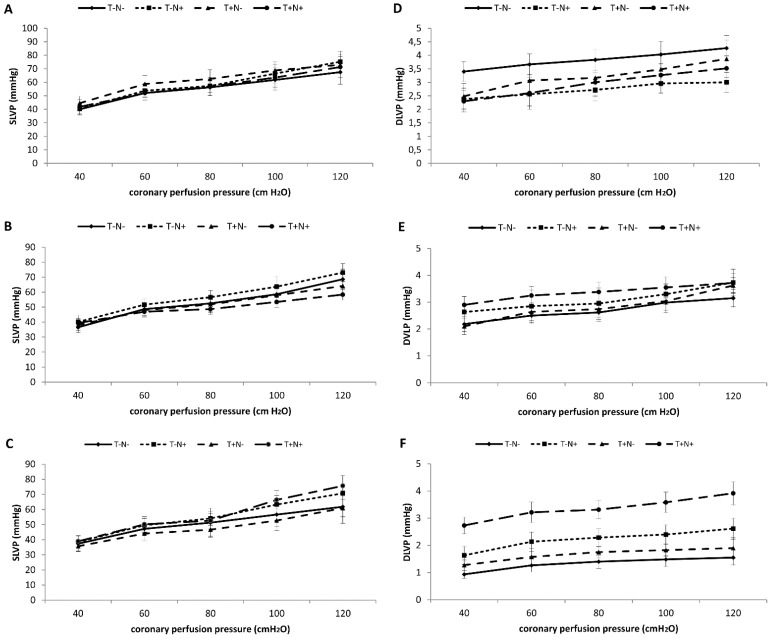
Mean values of SLVP (**A**–**C**) and DLVP (**D**–**F**). The comparation of all experimental groups after the second (**A**,**D**), third (**B**,**E**), and fourth (**C**,**F**) week of the experimental period. Each value represents the mean ± SD (*n* = 6).

Training alone, after the second experimental week, increased the values of systolic left ventricle pressure at all perfusion pressures compared to the control group. Nandrolone alone or combined with training increased the value of SLVP at higher perfusion pressure starting from 80 cm H_2_O (Figure 2A). After the third week, nandrolone alone increased the value of SLVP on all perfusion pressures, while the combined administration of nandrolone and training decreased this parameter compared to the T-N- group at the end of the third experimental week (Figure 2B). After the fourth week, the administration of nandrolone alone or combined with training significantly increased SLVP at higher perfusion pressures compared to control. Training alone decreased this parameter compared to control (Figure 2C). The statistical significance between groups is presented in Table 3.

#### 3.2.4. Diastolic Left Ventricle Pressure—DLVP

After the second experimental week, diastolic left ventricle pressure was significantly decreased in all experimental groups compared to control (Figure 2D). After the third week, the values of the DLVP were increased in the T-N+ and T+N+ groups compared to control (Figure 2E). The values of the DLVP were increased in all experimental groups compared to control after the fourth experimental week. The largest increase was observed in the group, which was exposed to the combined administration of nandrolone and training. Swimming training did not significantly alter DLVP in all experimental weeks compared to control values (Figure 2F). The significance of the statistical differences between groups is presented in Table 3.

#### 3.2.5. Heart Rate—HR

**Figure 3:**
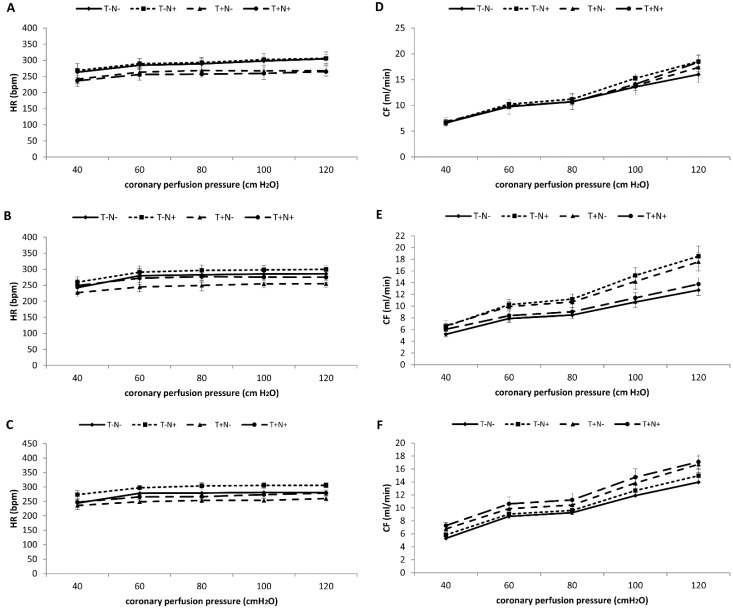
Mean values of heart rate (HR) (**A**–**C**) and coronary flow (CF) (**D**–**F**). Comparation of all experimental groups after the second (**A**,**D**), third (**B**,**E**), and fourth (**C**,**F**) week of the experimental period. Each value represents the mean ± SD (*n* = 6).

The values of the heart rate, after the second experimental week, were statistically significantly decreased in the T+N- and T+N+ groups compared to the T-N- group (Figure 3A). After the third week, training alone decreased this parameter significantly compared to control values (Figure 3B). After the fourth experimental week, HR in the nandrolone treated group was increased in all perfusion pressure values, while training alone decreased values on all perfusion pressure values compared to control values (Figure 3C). Statistical differences between groups were presented in Table 4.

#### 3.2.6. Coronary Flow—CF

After the two weeks of experimental protocol, nandrolone administration alone increased the value of coronary flow on all perfusion pressures, while training alone or combined with nandrolone increased CF at a higher perfusion pressure starting from 100 cm H_2_O compared to control (Figure 3D). The largest increase in coronary flow, after the third week, was observed in the nandrolone only and training only groups compared to the T-N- group (Figure 3E). As opposed to the third experimental week, in the fourth experimental week, the largest increase in this parameter was observed in the T+N+ group compared to control values (Figure 3F). The significance of the statistical differences between groups was presented in Table 4.

### 3.3. Morphometric Parameters of the Left Ventricle

#### 3.3.1. Longitudinal Section Diameter

**Figure 4:**
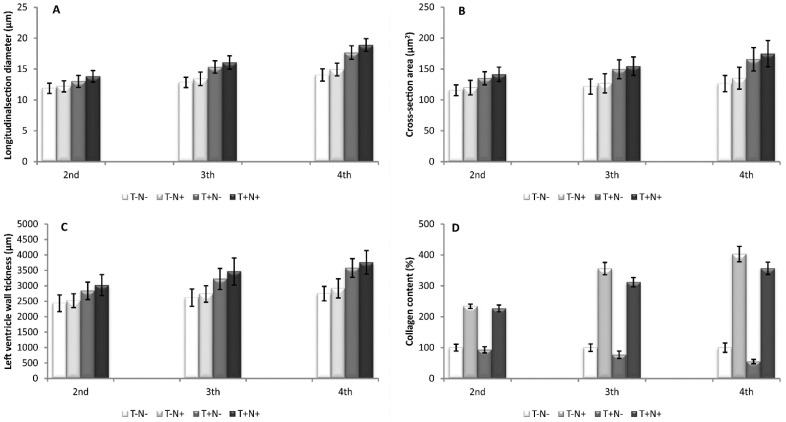
Longitudinal section diameter of the cardiomyocytes (**A**), cross-section area of the cardiomyocytes (**B**), left ventricle wall thickness (**C**), and collagen content (**D**) after the second, third, and fourth experimental week. Each value represents the mean ± SD (*n* = 6).

The longitudinal section diameter of the cardiomyocytes, after the second experimental week, was increased in the T+N- group by 10% and in the T+N+ group by 16.5% compared to control. In comparison to the experimental groups, swimming alone or combined with nandrolone increased the diameter of the cardiomyocyte by 7% and by 13%, respectively, compared to nandrolone alone. In the T+N+ group, the diameter was increased by 6% compared to the T+N- group. After the third experimental week, the diameter was increased in the T-N+ group by 4.5%, in the T+N- group by 19.5%, and in the T+N+ group by 25% in comparison to the control value. Swimming alone increased the diameter by 14% and the combined administration of nandrolone and swimming increased the diameter by 20% compared to nandrolone alone. The diameter of the cardiomyocyte after the fourth week was increased in the T+N- group by 25% and in the T+N+ group by 34.5% compared to the control. In the comparison between experimental groups, swimming alone or combined with nandrolone increased the diameter of the cardiomyocyte by 18% and by 27% compared to nandrolone alone. In the T+N+ group, the diameter was increased by 7% compared to swimming only (Figure 4A). The statistical significance between groups is presented in Table 5.

#### 3.3.2. Cross-Section Area

The cross-section area of the cardiomyocytes was increased after the second experimental week by 17% in the T+N- group and by 22% in the T+N+ group compared to control. In the T+N- and T+N+ groups, the area of the cardiomyocytes was increased by 12% and 18%, respectively, in comparison to the T-N+ group. After the third experimental week, the increase in the area of the cardiomyocytes in the T+N- group was 23% and 27% in the T+N+ group compared to control. In the comparison between experimental groups, swimming only or combined with nandrolone increased the cross-section area of cardiomyocytes by 16.5% and by 20.5%, respectively, compared to nandrolone alone. After the fourth week, the cross-section area of cardiomyocytes was increased in the T-N+ group by 7%, in the T+N- group by 30%, and in the T+N+ group by 38%. In the T+N- group and T+N+ group, the cross-section area was increased by 21% and 29%, respectively, compared to the T-N+ group (Figure 4B, Table 5).

#### 3.3.3. Left Ventricle Wall Thickness

After the second experimental week, the left ventricle wall thickness was increased by 16.5% in the T+N- group and by 24% in the T+N+ group. The increase of wall thickness in the T+N- group was 12.5%, and in the T+N+ group it was 20% compared to the T-N+ group. In the T+N+ group, the increase was 6.5% compared to the T+N- group. The wall thickness after the third experimental week was increased by 23% in the T+N- group and by 30% in the T+N+ group compared to the control value. In the comparison between experimental groups, swimming alone or combined with nandrolone increased the wall thickness by 18% and by 25.5%, respectively, in comparison to nandrolone alone. After the fourth experimental week, left ventricle wall thickness was increased by 6.5% in the T-N+ group, by 30% in the T+N- group, and by 37% in the T+N+ group in comparison to control. The % of increase in wall thickness was 22% in the T+N- group and 28% in the T+N+ group compared to the T-N+ group (Figure 4C, Table 5). 

#### 3.3.4. Collagen Content

**Figure 5:**
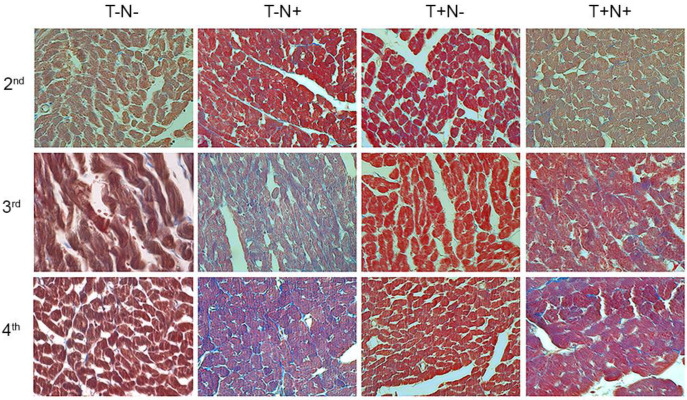
Masson trichrome staining of the cardiac muscle after two, three, and four weeks (objective magnification 40×, bar = 25 µm).

Collagen content in the cardiac muscle, after the second experimental week, was increased in the T-N+ group by 234% and in the T+N+ group by 227%, while in the T+N- group collagen was decreased by 7% compared to control values. After the third experimental week, the values were increased in the T-N+ group and in the T+N+ group by 356% and 312%, respectively, while in the T+N- group, values were decreased by 23% compared to the T-N-. After the fourth week, nandrolone administration alone or in combination with swimming increased collagen content by 403% and 357%, respectively, while swimming decreased collagen in cardiac muscle tissue by 45% compared to control (Figure 4D). Masson trichrome staining, presented in Figure 5, shows the blue deposits of the collagen fibers. The longer the experimental period, the coarser and more bundled the fibers/deposits in animals that received nandrolone become, especially in the T-N+ group but in the T+N+ group as well. Training alone, also shown above by image analysis, lowered the overall level of heart collagen. Statistical differences between groups were presented in Table 5.

## 4. Discussion

During the last few decades, nandrolone decanoate has become one of the most frequently abused AAS among professional and recreational sportsmen [21]. Regular physical activity, as a modern way of life, has positive effects on many organ systems, especially on the cardiovascular system and metabolic disorders [32]. To the best of our knowledge, this study is the first investigation into the effects of the time-dependent administration of nandrolone decanoate alone or combined with swimming training on the function of the isolated heart and morphometry of cardiomyocytes and collagen content in the left ventricle myocardium. 

In the present investigation, cardiac contractility (inotropic properties) as well as relaxation (lusitropic properties) was estimated by the maximum and minimum rate of left ventricle pressure development (dp/dt max, dp/dt min). After the initial adaptation, in the second and third week, the strength of the muscular contraction at the end of the experimental period was increased, which is a consequence of the combined effect of nandrolone decanoate and training. These results can be explained by the positive effect of training and the negative effect of nandrolone decanoate on the contractile ability of the myocardium but also by the influence of nandrolone on relaxation (diastole) of the hearts in the group that were receiving nandrolone through an increase in negative values of dp/dt min. Results from our study are consistent with those of other researchers, who have shown that AAS treatment leads to the dysfunction of autonomic regulation of the heart with the severe impairment of cardiac parasympathetic modulation, sympathetic hyperactivity, and changes in the sensitivity of the heart through the effect on β-adrenergic receptors, which results in a hypersensitivity sino-atrial node [33]. In their study, Melo Junior et al. showed that treatment with nandrolone alone led to an increase in the value of dp/dt max and dp/dt min, which correlates with our results [34]. A previous study has also shown that the reduction of myocardial relaxation represents one of the indications of heart failure [34]. 

The values of SLVP were increased in the nandrolone only group during the whole experiment. The increased values of SLVP and dp/dt max in nandrolone-treated rats are indicators of the systolic function of the heart. das Neves et al. showed that nandrolone alone or combined with training increased systolic blood pressure after the 3rd, 4th, and 5th week compared to control or training alone [35]. This result may be due to the reduction of NO (nitrogen oxide) production, which may be a consequence of nandrolone administration [36]. Additionally, the increased values in blood pressure caused by nandrolone administration can lead to the development of hypertrophy of the heart [35]. Bearing this in mind, we believe that the reason for the largest increase in SLVP in the group with the combined administration of nandrolone and swimming at the end of the fourth experimental week was the hypertrophy of cardiomyocytes, which is confirmed in this study by increase in the longitudinal section diameter and cross-section area of the cardiomyocytes as well as the left ventricle wall thicknesses.

After the initial period of adaptation in the second week, diastolic pressure was decreased in all experimental groups, while during the third and fourth week, diastolic function was increased in all experimental groups, especially in the group which received nandrolone decanoate in combination with training. In our study, the rate of myocardial relaxation was lower in the group that received nandrolone decanoate and, consequently, DLVP increased in the third and fourth experimental weeks. In this case, a smaller volume of blood flows into the left ventricle during diastole, and any filling impairment could compromise systolic function. The exact mechanism by which nandrolone affects this process is unknown to us. We can only assume that the hypertrophy of the heart could be one of the reasons that would compromise the relaxation of the heart, especially the increased content of collagen due to nandrolone administration.

Interestingly, after the second experimental week, the diastolic left ventricle pressure was significantly decreased in all experimental groups compared to control. During that period, cardiomyocytes hypertrophy started, which is shown as a slight increase of its longitudinal section diameter and cross-section area (Figure 4) as well as collagen content in the heart treated by nandrolone decanoate. Because of that, it would be possible that increased heart contractility could overcome the effect that small initial hypertrophy has by slowing ventricular relaxation. In that case, stroke volume increases in the heart and end-systole volume decreases, which consequently produces decreased DLVP. On the other hand, Shirpoor et al. showed that nandrolone alone or combined with swimming increase diastolic pressure [36], but the mechanism involved in this process is not yet understood. The literature data suggest that the mechanism, which involves changes in arterial blood pressure are cardiac hypertrophy, sodium balance changes, vascular degenerative lesions, and an unbalanced lipid profile [36,37,38]. 

The reduction of heart rate in swimming training and swimming training with nandrolone groups was observed during the 2nd, 3rd, and 4th experimental weeks. It occurred as a result of the swimming training process. The reduction of the heart rate is a consequence of adaptation in autonomic nervous activity in the heart induced by training [3]. Previous studies showed that swimming [3] and resistance training [7,35] induced bradycardia in rest, which represents a confirmation of the efficiency of the training process [7]. In our study, nandrolone treatment alone increased heart rate in the 3rd and 4th experimental week, which is in line with previous findings [3]. This is a consequence of increased sympathetic activity [3]. It should also be kept in mind that increased DLVP leads to a reduced filling of the left ventricle with blood and the heart compensates for this reduced inflow by increasing the heart rate. As opposed to this, Rocha et al. [7] as well as Melo Junior et al. [34] showed that treatment with nandrolone induced lower values of the heart rate compared to control. Combined treatment with nandrolone and swimming training decreased the value of the heart rate, which is in line with the results from other studies [7,35]. Contrary to this, a previous study showed that the combined treatment increased heart rate, which indicates that nandrolone blocks autonomic activity in the heart [3]. 

The largest increase of the left ventricle wall thickness was observed after the combined administration of nandrolone and training, which shows that training has the greatest impact on the occurrence of the thickening of the left ventricular wall. This result is in line with results from a previous study [14]. We know that the main reason for the increase in the left ventricle wall thickness is the hypertrophy of the cardiomyocytes in groups that were exposed to swimming, which is confirmed in our study (cross-section area and longitudinal section diameter (Figure 4)). On the other hand, scientists believe that the degree of the increase in cardiac wall thickness depends on the dose of the steroid and the duration of its administration [13], which have been shown in this study (an increase of 3.5% after the 2nd, 4.5% after the 3rd, and 6.5% after the 4th week). Even if high doses of nandrolone were used alone, it would not lead to a significant increase in wall thickness. 

The diameter of the cardiomyocytes, as well as the cross-section area, were increased after the administration of nandrolone decanoate. This result is in line with the literature data [14,34]. AAS induces the hypertrophy of cardiomyocytes by increasing the incorporation of amino acids into the protein [39]. On the other hand, nandrolone decanoate in skeletal muscle promotes the delay of nitrogen and in that way leads to an increase in skeletal muscle hypertrophy [11]. We assume that this mechanism is possibly responsible for the hypertrophy of cardiomyocytes after nandrolone treatment. Contrary to this, Rocha et al. reported that nandrolone alone, administered over a period of ten weeks, did not increase the diameter of cardiomyocytes [7]. Swimming alone or combined with nandrolone increased the cross-section area and the diameter of cardiomyocytes, caused by the physiological hypertrophy of cardiomyocytes induced by swimming. This result is in accordance with literature data [7,14,40]. On the other hand, Locanelli et al. showed that swimming decreased cardiomyocyte thickness in SHR (spontaneously hypertensive rats) rats [40]. The reason for this result lies in the fact that swimming likely leads to increased and decreased angiotensin (1-7) [40]. 

In our study, nandrolone alone or combined with swimming increased collagen content in every experimental week, especially in the 4th week, which is in line with literature data [7,14,23]. This result showed that a higher content of collagen led to the development of cardiac fibrosis, which can be followed by diastolic and systolic dysfunction [7]. The administration of androgens has a significant impact on the remodeling of the left ventricle heart [41,42]. Data from the literature indicate that the increase in collagen content in the heart as well as the presence of cardiac hypertrophy, together with RAS (renin angiotensin system) and TNF-α (tumor necrosis factor alpha) represent an important component for the development of cardiac remodeling [34,43], resulting in a decreased contractile capacity of the heart and increased heart tenseness [34], which is also confirmed in our study taking into account the increase in dp/dt max and decrease in dp/dt min as a marker of cardiodynamic properties. Interesting data were obtained regarding collagen content after swimming training. Collagen content decreased after every experimental week, which is in accordance with literature data [12,40,44]. This result is a consequence of the positive effects of swimming on the myocardium. 

Unfortunately, there is insufficient information in the available literature on the mechanism that is responsible for the reduction of collagen content in the cardiac muscle after swimming training. Locanelli et al. showed that swimming decreased collagen content in Wistar and SHR rats [40]. They stated that the decrease in collagen in the LV (left ventricle) in SHR rats results from the fact that angiotensin (1-7) causes the inhibition of collagen synthesis [40]. In contrast to our results, Tanno showed that resistance training increased collagen content in cardiac muscle and consequently led to the development of the pathological hypertrophy of the heart [23]. Obviously, the collagen content in cardiac muscle tissue depends on the type of physical activity to which the individual is exposed. The increase in collagen content was observed in the group that was exposed to the combined administration of nandrolone and swimming, which is in accordance with the literature [7,23,45]. Results showed that resistance training combined with nandrolone leads to the development of hypertrophy of cardiomyocytes, increasing collagen content in cardiac muscle, systolic, and diastolic dysfunction, which confirms the occurrence of the pathological hypertrophy of the left heart [23,45]. This result suggests that the combined use of nandrolone and training have a synergistic effect on the enlargement of the left ventricular myocardium, which may result in cardiac dysfunction [23,45].

The findings of this study have to be seen in the light of some limitations. Firstly, despite being commonly employed as an animal model for research, rats do not have similar abilities to humans regarding exercise. To demonstrate whether there are histological or molecular alterations related to nandrolone decanoate and exercise additional methods of study should be performed.

## 5. Conclusions

Generally, we can conclude that the supraphysiological doses of nandrolone decanoate alone or combined with swimming training, even if used in a short-term period, have a deleterious effect on myocardial function and perfusion. On the other hand, swimming itself improved cardiac function and perfusion. However, swimming alone increases cardiac muscle hypertrophy and decreases collagen content in cardiac muscle. We believe that further investigations are needed to assess the mechanism responsible for decreased collagen content in rats exposed to swimming. 

## Figures and Tables

**Table 1:** Average values of body weight (BW), heart weight (HW), and heart weight/body weight ratio (HW/BW).

Groups	2nd Week			3rd Week			4th Week		
	BW (g)	HW (g)	HW/BW (%)	BW (g)	HW (g)	HW/BW (%)	BW (g)	HW (g)	HW/BW (%)
**T-N-**	367.33 ± 12.61	1.55 ± 0.10	0.47 ± 0.02	400.5 ± 13.03	1.65 ± 0.05	0.41 ± 0.01	431 ± 14.84	1.77 ± 0.08	0.41 ± 0.01
**T-N+**	358 ± 15.79	1.5 ± 0.09 **d,e**	0.42 ± 0.04 **a,d,e**	382.83 ± 17.76 **a**	1.6 ± 0.10	0.42 ± 0.04	418.67 ± 17.52	1.67 ± 0.08	0.40 ± 0.01
**T+N-**	353 ± 11.96	1.67 ± 0.10 **b,d**	0.50 ± 0.02 **d**	385.17 ± 12.18	1.75 ± 0.05 **d**	0.45 ± 0.02 **b**	421.17 ± 16.57	1.88 ± 0.09	0.48 ± 0.01
**T+N+**	351.33 ± 9.99 **c**	1.78 ± 0.07 **c,e,f**	0.51 ± 0.03 **c,e**	381.33 ± 11.84 **c**	1.72 ± 0.11 **c,e**	0.45 ± 0.04 **c**	419.33 ± 17.52	1.93 ± 0.10	0.46 ± 0.01

Results presented as mean value ± SD (*n* = 6). Statistical analysis was performed using a one way ANOVA test with the post hoc LSD test analysis. The bold represents the abbreviated name of groups, the parameter that was monitored in each experimental week, as well as the statistically significant change of the investigated parameter. Statistical significance between groups is shown as a, b, c, d, e, f: a denotes T-N+ vs. T-N-; b denotes T+N- vs. T-N-; c denotes T+N+ vs. T-N-; d denotes T+N- vs. T-N+; e denotes T+N+ vs. T-N+; f denotes T+N+ vs. T+N-; T-N- (control), T-N+ (nandrolone), T+N- (swimming training), and T+N+ (swimming training plus nandrolone).

**Table 2:** Significance of the statistical difference in the values of the dp/dt max and dp/dt min between groups after the 2nd, 3rd, and 4th experimental week at different values of coronary perfusion pressures.

**2nd Experimental Week**										
**Groups**	**dp/dt max**					**dp/dt min**				
	**40**	**60**	**80**	**100**	**120**	**40**	**60**	**80**	**100**	**120**
**T-N+ vs. T-N-**	*p* > 0.05	*p* > 0.05	*p* > 0.05	***p* = 0.040**	***p* = 0.010**	*p* > 0.05	*p* > 0.05	*p* > 0.05	***p* = 0.037**	***p* = 0.015**
**T+N- vs. T-N-**	*p* > 0.05	***p* = 0.007**	***p* = 0.008**	*p* > 0.05	*p* > 0.05	***p* = 0.026**	***p* = 0.001**	***p* = 0.002**	***p* = 0.022**	***p* = 0.032**
**T+N+ vs. T-N-**	***p* = 0.048**	*p* > 0.05	*p* > 0.05	*p* > 0.05	*p* > 0.05	*p* > 0.05	*p* > 0.05	*p* > 0.05	*p* > 0.05	*p* > 0.05
**T+N- vs. T-N+**	*p* > 0.05	*p* > 0.05	*p* > 0.05	*p* > 0.05	*p* > 0.05	***p* = 0.014**	*p* = 0.001	***p* = 0.037**	*p* > 0.05	*p* > 0.05
**T+N+ vs. T-N+**	*p* > 0.05	*p* > 0.05	*p* > 0.05	***p* = 0.045**	***p* = 0.000**	*p* > 0.05	*p* > 0.05	*p* > 0.05	*p* > 0.05	*p* > 0.05
**T+N+ vs. T+N-**	***p* = 0.017**	***p* = 0.005**	***p* = 0.012**	*p* > 0.05	***p* = 0.002**	***p* = 0.021**	***p* = 0.003**	***p* = 0.006**	***p* = 0.047**	*p* > 0.05
**3rd Experimental Week**										
	**dp/dt max**					**dp/dt min**				
	**40**	**60**	**80**	**100**	**120**	**40**	**60**	**80**	**100**	**120**
**T-N+ vs. T-N-**	***p* = 0.002**	*p* > 0.05	***p* = 0.023**	***p* = 0.000**	***p* = 0.000**	*p* > 0.05	*p* > 0.05	*p* > 0.05	***p* = 0.003**	***p* = 0.004**
**T+N- vs. T-N-**	*p* > 0.05	*p* > 0.05	*p* > 0.05	*p* > 0.05	*p* > 0.05	*p* > 0.05	*p* > 0.05	*p* > 0.05	*p* > 0.05	*p* > 0.05
**T+N+ vs. T-N-**	***p* = 0.027**	*p* > 0.05	*p* > 0.05	*p* > 0.05	*p* > 0.05	***p* = 0.035**	*p* > 0.05	*p* > 0.05	*p* > 0.05	*p* > 0.05
**T+N- vs. T-N+**	***p* = 0.023**	*p* > 0.05	*p* > 0.05	***p* = 0.001**	***p* = 0.000**	*p* > 0.05	*p* > 0.05	*p* > 0.05	***p* = 0.004**	***p* = 0.001**
**T+N+ vs. T-N+**	*p* > 0.05	***p* = 0.017**	***p* = 0.004**	***p* = 0.000**	***p* = 0.000**	*p* > 0.05	*p* > 0.05	***p* = 0.025**	***p* = 0.000**	***p* = 0.001**
**T+N+ vs. T+N-**	*p* > 0.05	*p* > 0.05	*p* > 0.05	*p* > 0.05	*p* > 0.05	*p* > 0.05	*p* > 0.05	*p* > 0.05	*p* > 0.05	*p* > 0.05
**4th Experimental Week**										
	**dp/dt max**					**dp/dt min**				
	**40**	**60**	**80**	**100**	**120**	**40**	**60**	**80**	**100**	**120**
**T-N+ vs. T-N-**	*p* > 0.05	*p* > 0.05	*p* > 0.05	***p* = 0.007**	***p* = 0.002**	*p* > 0.05	*p* > 0.05	*p* > 0.05	*p* > 0.05	***p* = 0.020**
**T+N- vs. T-N-**	*p* > 0.05	*p* > 0.05	*p* > 0.05	*p* > 0.05	*p* > 0.05	*p* > 0.05	***p* = 0.021**	*p* > 0.05	***p* = 0.032**	***p* = 0.023**
**T+N+ vs. T-N-**	*p* > 0.05	*p* > 0.05	*p* > 0.05	*p* > 0.05	***p* = 0.007**	*p* > 0.05	*p* > 0.05	*p* > 0.05	*p* > 0.05	*p* > 0.05
**T+N- vs. T-N+**	***p* = 0.042**	***p* = 0.008**	***p* = 0.006**	***p* = 0.001**	***p* = 0.002**	*p* > 0.05	***p* = 0.002**	***p* = 0.004**	***p* = 0.001**	***p* = 0.000**
**T+N+ vs. T-N+**	*p* > 0.05	***p* = 0.016**	***p* = 0.032**	*p* > 0.05	*p* > 0.05	*p* > 0.05	*p* > 0.05	*p* > 0.05	*p* > 0.05	*p* > 0.05
**T+N+ vs. T+N-**	*p* > 0.05	*p* > 0.05	*p* > 0.05	***p* = 0.017**	***p* = 0.007**	*p* > 0.05	*p* > 0.05	*p* > 0.05	***p* = 0.005**	***p* = 0.001**

Statistical analysis was carried out using one way ANOVA test with the post hoc LSD test analysis. The bold represents the abbreviated name of groups, the parameter that was monitored in each experimental week, as well as the statistically significant change of the investigated parameter. dp/dt max (maximum rate of pressure development); dp/dt min (minimum rate of pressure development); T-N- (control); T-N+ (nandrolone); T+N- (swimming training); T+N+ (swimming training plus nandrolone).

**Table 3:** Significance of the statistical difference in the values of the SLVP and DLVP between groups after the 2nd, 3rd, and 4th experimental week at different values of coronary perfusion pressures.

**2nd Experimental Week**										
**Groups**	**SLVP**					**DLVP**				
	**40**	**60**	**80**	**100**	**120**	**40**	**60**	**80**	**100**	**120**
**T-N+ vs. T-N-**	*p* > 0.05	*p* > 0.05	*p* > 0.05	*p* > 0.05	*p* > 0.05	***p* = 0.001**	***p* = 0.003**	***p* = 0.001**	***p* = 0.001**	***p* = 0.001**
**T+N- vs. T-N-**	*p* > 0.05	***p* = 0.030**	*p* > 0.05	*p* > 0.05	*p* > 0.05	***p* = 0.001**	*p* > 0.05	***p* = 0.027**	*p* > 0.05	*p* > 0.05
**T+N+ vs. T-N-**	*p* > 0.05	*p* > 0.05	*p* > 0.05	*p* > 0.05	*p* > 0.05	***p* = 0.000**	***p* = 0.002**	***p* = 0.008**	***p* = 0.011**	***p* = 0.021**
**T+N- vs. T-N+**	*p* > 0.05	*p* > 0.05	*p* > 0.05	*p* > 0.05	*p* > 0.05	*p* > 0.05	*p* > 0.05	*p* > 0.05	*p* > 0.05	***p* = 0.012**
**T+N+ vs. T-N+**	*p* > 0.05	*p* > 0.05	*p* > 0.05	*p* > 0.05	*p* > 0.05	*p* > 0.05	*p* > 0.05	*p* > 0.05	*p* > 0.05	*p* > 0.05
**T+N+ vs. T+N-**	*p* > 0.05	***p* = 0.031**	*p* > 0.05	*p* > 0.05	*p* > 0.05	*p* > 0.05	*p* > 0.05	*p* > 0.05	*p* > 0.05	*p* > 0.05
**3rd Experimental Week**										
	**SLVP**					**DLVP**				
	**40**	**60**	**80**	**100**	**120**	**40**	**60**	**80**	**100**	**120**
**T-N+ vs. T-N-**	*p* > 0.05	*p* > 0.05	*p* > 0.05	*p* > 0.05	*p* > 0.05	***p* = 0.022**	*p* > 0.05	*p* > 0.05	*p* > 0.05	***p* = 0.013**
**T+N- vs. T-N-**	*p* > 0.05	*p* > 0.05	*p* > 0.05	*p* > 0.05	*p* > 0.05	*p* > 0.05	*p* > 0.05	*p* > 0.05	*p* > 0.05	***p* = 0.049**
**T+N+ vs. T-N-**	*p* > 0.05	*p* > 0.05	*p* > 0.05	*p* > 0.05	***p* = 0.012**	***p* = 0.001**	***p* = 0.000**	***p* = 0.004**	***p* = 0.012**	***p* = 0.015**
**T+N- vs. T-N+**	*p* > 0.05	*p* > 0.05	*p* > 0.05	*p* > 0.05	***p* = 0.033**	***p* = 0.011**	*p* > 0.05	*p* > 0.05	*p* > 0.05	*p* > 0.05
**T+N+ vs. T-N+**	*p* > 0.05	*p* > 0.05	***p* = 0.014**	***p* = 0.006**	***p* = 0.001**	*p* > 0.05	***p* = 0.037**	*p* > 0.05	*p* > 0.05	*p* > 0.05
**T+N+ vs. T+N-**	*p* > 0.05	*p* > 0.05	*p* > 0.05	*p* > 0.05	*p* > 0.05	***p* = 0.000**	***p* = 0.004**	***p* = 0.016**	***p* = 0.027**	*p* > 0.05
**4th experimental week**										
	**SLVP**					**DLVP**				
	**40**	**60**	**80**	**100**	**120**	**40**	**60**	**80**	**100**	**120**
**T-N+ vs. T-N-**	*p* > 0.05	*p* > 0.05	*p* > 0.05	***p* = 0.047**	***p* = 0.021**	***p* = 0.000**	***p* = 0.000**	***p* = 0.000**	***p* = 0.000**	***p* = 0.000**
**T+N- vs. T-N-**	*p* > 0.05	*p* > 0.05	*p* > 0.05	*p* > 0.05	*p* > 0.05	***p* = 0.040**	*p* > 0.05	*p* > 0.05	*p* > 0.05	*p* > 0.05
**T+N+ vs. T-N-**	*p* > 0.05	*p* > 0.05	*p* > 0.05	***p* = 0.005**	***p* = 0.001**	***p* = 0.000**	***p* = 0.000**	***p* = 0.000**	***p* = 0.000**	***p* = 0.000**
**T+N- vs. T-N+**	*p* > 0.05	*p* > 0.05	***p* = 0.023**	***p* = 0.005**	***p* = 0.016**	***p* = 0.037**	***p* = 0.011**	***p* = 0.008**	***p* = 0.006**	***p* = 0.005**
**T+N+ vs. T-N+**	*p* > 0.05	*p* > 0.05	*p* > 0.05	*p* > 0.05	*p* > 0.05	***p* = 0.000**	***p* = 0.000**	***p* = 0.000**	***p* = 0.000**	***p* = 0.000**
**T+N+ vs. T+N-**	*p* > 0.05	***p* = 0.045**	*p* > 0.05	***p* = 0.001**	***p* = 0.001**	***p* = 0.000**	***p* = 0.000**	***p* = 0.000**	***p* = 0.000**	***p* = 0.000**

Statistical analysis was performed using one way ANOVA test with post hoc LSD test analysis. The bold represents the abbreviated name of groups, the parameter that was monitored in each experimental week, as well as the statistically significant change of the investigated parameter. SLVP (systolic left ventricle pressure); DLVP (diastolic left ventricle pressure); T-N- (control); T-N+ (nandrolone); T+N- (swimming training); T+N+ (swimming training plus nandrolone).

**Table 4:** Significance of the statistical difference in the values of the HR and CF between groups after the 2nd, 3rd, and 4th experimental week at different values of coronary perfusion pressures.

**2nd Experimental Week**										
**Groups**	**HR**					**CF**				
	**40**	**60**	**80**	**100**	**120**	**40**	**60**	**80**	**100**	**120**
**T-N+ vs. T-N-**	*p* > 0.05	*p* > 0.05	*p* > 0.05	*p* > 0.05	*p* > 0.05	*p* > 0.05	*p* > 0.05	*p* > 0.05	*p* > 0.05	*p* > 0.05
**T+N- vs. T-N-**	***p* = 0.037**	***p* = 0.022**	***p* = 0.024**	***p* = 0.005**	***p* = 0.001**	*p* > 0.05	*p* > 0.05	*p* > 0.05	*p* > 0.05	*p* > 0.05
**T+N+ vs. T-N-**	***p* = 0.010**	***p* = 0.003**	***p* = 0.001**	***p* = 0.001**	***p* = 0.000**	*p* > 0.05	*p* > 0.05	*p* > 0.05	*p* > 0.05	*p* > 0.05
**T+N- vs. T-N+**	***p* = 0.015**	***p* = 0.009**	***p* = 0.011**	***p* = 0.003**	***p* = 0.001**	*p* > 0.05	*p* > 0.05	*p* > 0.05	*p* > 0.05	*p* > 0.05
**T+N+ vs. T-N+**	***p* = 0.004**	***p* = 0.001**	***p* = 0.001**	***p* = 0.001**	***p* = 0.000**	*p* > 0.05	*p* > 0.05	*p* > 0.05	*p* > 0.05	*p* > 0.05
**T+N+ vs. T+N-**	*p* > 0.05	*p* > 0.05	*p* > 0.05	*p* > 0.05	*p* > 0.05	*p* > 0.05	*p* > 0.05	*p* > 0.05	*p* > 0.05	*p* > 0.05
**3rd Experimental Week**										
	**HR**					**CF**				
	**40**	**60**	**80**	**100**	**120**	**40**	**60**	**80**	**100**	**120**
**T-N+ vs. T-N-**	*p* > 0.05	*p* > 0.05	*p* > 0.05	*p* > 0.05	*p* > 0.05	***p* = 0.004**	***p* = 0.001**	***p* = 0.000**	***p* = 0.001**	***p* = 0.000**
**T+N- vs. T-N-**	*p* > 0.05	***p* = 0.008**	*p* = 0.006	***p* = 0.005**	***p* = 0.004**	***p* = 0.001**	***p* = 0.001**	***p* = 0.000**	***p* = 0.000**	***p* = 0.000**
**T+N+ vs. T-N-**	*p* > 0.05	*p* > 0.05	*p* > 0.05	*p* > 0.05	*p* > 0.05	***p* = 0.030**	*p* > 0.05	*p* > 0.05	*p* > 0.05	*p* > 0.05
**T+N- vs. T-N+**	***p* = 0.004**	***p* = 0.001**	***p* = 0.000**	***p* = 0.000**	***p* = 0.000**	*p* > 0.05	*p* > 0.05	*p* > 0.05	*p* > 0.05	*p* > 0.05
**T+N+ vs. T-N+**	*p* > 0.05	*p* > 0.05	*p* > 0.05	***p* = 0.027**	***p* = 0.014**	*p* > 0.05	***p* = 0.008**	***p* = 0.005**	***p* = 0.008**	***p* = 0.002**
**T+N+ vs. T+N-**	***p* = 0.035**	***p* = 0.033**	***p* = 0.021**	***p* = 0.045**	***p* = 0.047**	*p* > 0.05	***p* = 0.006**	***p* = 0.003**	***p* = 0.001**	***p* = 0.000**
**4th Experimental Week**										
	**HR**					**CF**				
	**40**	**60**	**80**	**100**	**120**	**40**	**60**	**80**	**100**	**120**
**T-N+ vs. T-N-**	***p* = 0.000**	***p* = 0.003**	***p* = 0.000**	***p* = 0.001**	***p* = 0.000**	*p* > 0.05	*p* > 0.05	*p* > 0.05	*p* > 0.05	*p* > 0.05
**T+N- vs. T-N-**	*p* > 0.05	***p* = 0.000**	***p* = 0.000**	***p* = 0.000**	***p* = 0.001**	***p* = 0.001**	***p* = 0.041**	***p* = 0.038**	***p* = 0.014**	***p* = 0.001**
**T+N+ vs. T-N-**	*p* > 0.05	***p* = 0.027**	***p* = 0.040**	*p* > 0.05	*p* > 0.05	***p* = 0.000**	***p* = 0.001**	***p* = 0.001**	***p* = 0.001**	***p* = 0.000**
**T+N- vs. T-N+**	***p* = 0.000**	***p* = 0.000**	***p* = 0.000**	***p* = 0.000**	***p* = 0.000**	***p* = 0.019**	*p* > 0.05	*p* > 0.05	*p* > 0.05	***p* = 0.020**
**T+N+ vs. T-N+**	***p* = 0.001**	***p* = 0.000**	***p* = 0.000**	***p* = 0.000**	***p* = 0.000**	***p* = 0.001**	***p* = 0.007**	***p* = 0.004**	***p* = 0.007**	***p* = 0.004**
**T+N+ vs. T+N-**	*p* > 0.05	***p* = 0.007**	***p* = 0.047**	***p* = 0.004**	***p* = 0.003**	*p* > 0.05	*p* > 0.05	*p* > 0.05	*p* > 0.05	*p* > 0.05

Statistical analysis was performed using a one way ANOVA test with post hoc LSD test analysis. The bold represents the abbreviated name of groups, the parameter that was monitored in each experimental week, as well as the statistically significant change of the investigated parameter. HR (heart rate); CF (coronary flow); T-N- (control); T-N+ (nandrolone); T+N- (swimming training); T+N+ (swimming training plus nandrolone).

**Table 5:** Significance of the statistical difference in the values of the longitudinal section diameter, cross-section area, left ventricle wall thickness, and collagen content between groups after the 2nd, 3rd, and 4th experimental week.

Groups	T-N+ vs. T-N-	T+N- vs. T-N-	T+N+ vs. T-N-	T+N- vs. T-N+	T+N+ vs. T-N+	T+N+ vs. T+N-
**2nd Experimetal Week**						
**Longitudinal section diameter**	*p* > 0.05	***p* = 0.000**	***p* = 0.000**	***p* = 0.001**	***p* = 0.000**	***p* = 0.005**
**Cross-section area**	*p* > 0.05	***p* = 0.000**	***p* = 0.000**	***p* = 0.000**	***p* = 0.000**	*p* > 0.05
**Left ventricle wall thickness**	*p* > 0.05	***p* = 0.000**	***p* = 0.000**	***p* = 0.001**	***p* = 0.000**	*p* > 0.05
**Collagen content**	***p* = 0.000**	*p* > 0.05	***p* = 0.000**	***p* = 0.000**	*p* > 0.05	***p* = 0.000**
**3rd Experimental Week**						
**Longitudinal section diameter**	*p* > 0.05	***p* = 0.000**	***p* = 0.000**	***p* = 0.000**	***p* = 0.000**	***p* = 0.033**
**Cross-section area**	*p* > 0.05	***p* = 0.000**	***p* = 0.000**	***p* = 0.000**	***p* = 0.000**	*p* > 0.05
**Left ventricle wall thickness**	*p* > 0.05	***p* = 0.000**	***p* = 0.000**	***p* = 0.000**	***p* = 0.000**	*p* > 0.05
**Collagen content**	***p* = 0.000**	***p* = 0.019**	***p* = 0.000**	***p* = 0.000**	***p* = 0.000**	***p* = 0.000**
**4th Experimental Week**						
**Longitudinal section diameter**	***p* = 0.013**	***p* = 0.000**	***p* = 0.000**	***p* = 0.000**	***p* = 0.000**	***p* = 0.001**
**Cross-section area**	*p* > 0.05	***p* = 0.000**	***p* = 0.000**	***p* = 0.000**	***p* = 0.000**	*p* > 0.05
**Left ventricle wall thickness**	*p* > 0.05	***p* = 0.000**	***p* = 0.000**	***p* = 0.000**	***p* = 0.000**	*p* > 0.05
**Collagen content**	***p* = 0.000**	***p* = 0.000**	***p* = 0.000**	***p* = 0.000**	***p* = 0.000**	***p* = 0.000**

Statistical analysis was carried out using a one way ANOVA test with post hoc LSD test analysis. The bold represents the abbreviated name of groups, the parameter that was monitored in each experimental week, as well as the statistically significant change of the investigated parameter. T-N- (control); T-N+ (nandrolone); T+N- (swimming training); T+N+ (swimming training plus nandrolone).

## Data Availability

Not applicable.
